# Nitrogen Removal Characteristics and Constraints of an *Alphaproteobacteria* with Potential for High Nitrogen Content Heterotrophic Nitrification-Aerobic Denitrification

**DOI:** 10.3390/microorganisms10020235

**Published:** 2022-01-21

**Authors:** Nan Zhang, Yiting Zhang, Tsing Bohu, Shanghua Wu, Zhihui Bai, Xuliang Zhuang

**Affiliations:** 1 Key Laboratory of Environmental Biotechnology, Research Center for Eco-Environmental Sciences, Chinese Academy of Sciences, Beijing 100085, China; nanzhang@rcees.ac.cn (N.Z.); Zhangyiting0207123@163.com (Y.Z.); shwu@rcees.ac.cn (S.W.); zhbai@rcees.ac.cn (Z.B.); 2 Sino-Danish College, University of Chinese Academy of Sciences, Beijing 100049, China; 3 School of Environmental Science and Engineering, Hebei University of Science and Technology, Shijiazhuang 050018, China; 4 State Key Laboratory of Lunar and Planetary Sciences, Macau University of Science and Technology, Taipa, Macao; 5 CNSA Macau Center for Space Exploration and Science, Taipa, Macao; 6 CSIRO Mineral Resources, Australian Resources and Research Centre, Kensington, WA 6151, Australia; 7 Xiongan Institute of Innovation, Xiongan New Area, Baoding 071000, China; 8 Institute of Tibetan Plateau Research, Chinese Academy of Sciences, Beijing 100101, China

**Keywords:** aerobic, denitrification, heterotrophic, nitrification, *Pannonibacter*, wastewater

## Abstract

The discovery of heterotrophic nitrification-aerobic denitrification (HN-AD) microorganisms has opened a new window for wastewater treatment. The underlying mechanism of HN-AD, however, is not fully understood because of the phylogenetic diversity of HN-AD microbes. The isolation and characterization of new HN-AD microorganisms are encouraging for furthering the understanding of this process. In this study, we found an *Alphaproteobacteria* isolate W30 from a historically polluted river in China through an HN-AD microbes screening process, which we identified as *Pannonibacter* sp. A potential HN-AD pathway for W30 was proposed based on N conversion analyses and the successful amplification of the entire denitrification gene series. The isolate exhibited high efficiency of aerobic inorganic nitrogen transformation, which accounted for 97.11% of NH_4_^+^-N, 100% of NO_3_^−^-N, and 99.98% of NO_2_^−^-N removal with a maximum linear rate of 10.21 mg/L/h, 10.46 mg/L/h, and 10.77 mg/L/h, respectively. Assimilation rather than denitrification was the main mechanism for the environmental nitrogen depletion mediated by W30. The effect of environmental constraints on aerobic NO_3_^−^-N removal were characterized, following a membrane bioreactor effluent test under an oxic condition. Compared to known Alphaproteobacterial HN-AD microbes, we showed that *Pannonibacter* sp. W30 could deplete nitrogen with no NO_2_^−^-N or NO_3_^−^-N accumulation in the HN-AD process. Therefore, the application of *Pannonibacter* sp. W30 has the potential for developing a felicitous HN-AD technology to treat N-laden wastewater at the full-scale level.

## 1. Introduction

Traditional biological nitrogen removal technologies usually involve a two-step process with different types of microbes, autotrophic nitrifying bacteria, and denitrifiers (reducing NO_3_^−^-N or NO_2_^−^-N to gaseous nitrogen) [1]. Alternately, the heterotrophic nitrification-aerobic denitrification (HN-AD) process achieves simultaneous nitrification and denitrification in a system [2].

Heavy organic and inorganic nitrogen (N) pollution in water bodies is a burgeoning environmental issue worldwide [3,4,5,6]. Toggling between oxic and anoxic conditions, the traditional biological wastewater treatments that started from autotrophic nitrification (NH_4_^+^-N → NO_2_^−^-N → NO_3_^−^-N) compromise to low efficiency and incomplete N removal [1]. The development of heterotrophic nitrification-aerobic denitrification (HN-AD) processes, on the other hand, offers an alternate “one-pot” solution for wastewater treatment [2]. It has been well-recognized that HN-AD has several advantages over traditional methods in terms of carbon utilization, alkalinity demand, and space and energy requirements [7].

Fueled by organic carbons, microbes in HN-AD processes can simultaneously transform NH_4_^+^-N to NO_3_^−^-N and NO_2_^−^-N, and use O_2_, NO_3_^−^-N, and NO_2_^−^-N as the electron acceptors [8,9]. However, HN-AD encounters the same challenge found in traditional processes when tackling high N content wastewater. High N content in wastewater exerts at least two undesirable effects. On one hand, the removal of N from domestic wastewater with high N content can be limited by the fact that heterotrophic denitrifiers need sufficient carbon sources as electron donors. At the industry level, the removal of N from domestic wastewater with a low C/N ratio can also be limited by the fact that heterotrophic denitrifiers need sufficient carbon sources as electron donors [10]. Therefore, external carbon amendment is a common resort for treating this type of domestic wastewater in practice, leading to an extra carbon resource requirement. On the other hand, depending on functional genes and metabolism pathways, denitrification with high N content can sometimes accumulate nitrite, which inhibits the activity of denitrifying bacteria in wastewater treatments [11].

HN-AD microbes are ubiquitous and can prevail in activated sludge [12,13], aquaculture wastewater [14], marine sediments [15] and other different ecological environments. Microbes involved in HN-AD belong to various taxonomic groups, therefore their intra-group diversity is wide. Known HN-AD bacteria are included but not limited to *Paracoccus denitrificans* [16], *Rhodococcus* sp. [17], *Pseudomonas* sp. [18,19,20], *Bacillus* sp. [3,21], *Alcaligenes* sp. [22], and *Halomonas* sp. [23]. Among the known HN-AD microbes, only a few belong to the class of *Alphaproteobacteria*. Since most *Alphaproteobacteria* are oligotrophs that may adapt to nutrient variation in the environment, these microbes may apply novel mechanisms to tackle the low C/N ratio issue in HN-AD [24].

In this study, we screened a historically eutrophic domestic river in Beijing, China, for isolating aerobic nitrogen removal bacteria. Isolate W30 was identified as *Pannonibacter* sp., which belongs to *Alphaproteobacteria*. Its HN-AD capability was investigated using NH_4_^+^-N, NO_3_^−^-N, and NO_2_^−^-N as sole nitrogen sources, respectively. Based on N transformation assay and functional genes amplification, we suggest that isolate W30 is capable of carrying out nitrification and denitrification pathways. Since aerobic denitrification is the critical step for HN-AD, the species-specific N removal constraints were investigated. These factors were C/N ratio, carbon resource, dissolved oxygen, initial pH, temperature, and inoculation volume. Subsequently, the feasibility of isolate W30 in treating high N content wastewater at the full-scale level was tested using the effluent of a pilot membrane bioreactor (MBR). Our results suggest that (1) *Pannonibacter* sp. W30 is able to conduct HN-AD; (2) oxygen, however, is still a limiting factor affecting aerobic denitrification mediated by W30; (3) *Pannonibacter* sp. W30 adapts to a broad range of C/N ratios in wastewaters.

## 2. Materials and Methods

### 2.1. Media

Enrichment medium (EM): The EM was composed of 0.5 g/L (NH_4_)_2_SO_4_, 0.36 g/L KNO_3_, 4.0 g/L sodium citrate, and 0.05% (ratio of volume) of the trace element solution which was composed of 6.5 g/L K_2_HPO_4_·3H_2_O, 2.5 g/L MgSO_4_·7H_2_O, 2.5 g/L NaCl, 0.05 g/L FeSO_4_·7H_2_O, and 0.04 g/L MnSO_4_·H_2_O. The final pH of the EM was adjusted to 7.0.

Denitrifying medium (DM): The DM was composed of 0.36 g/L KNO_3_, 10.55 g/L Na_2_HPO_4_·12H_2_O, 1.5 g/L KH_2_PO_4_, 0.1 g/L MgSO_4_·7H_2_O, 4.0 g/L sodium citrate, and 0.2% (volume ratio) of trace element solution which included 50.0 g/L EDTA-Na_2_, 2.2 g/L ZnSO_4_, 5.5 g/L CaCl_2_, 5.06 g/L MnCl_2_·4H_2_O, 5.0 g/L FeSO_4_·7H_2_O, 1.57 g/L CuSO_4_·5H_2_O, and 1.61 g/L CoCl_2_·6H_2_O. The final pH of the DM was adjusted to 7.0.

Screen medium (GN): The GN for the denitrifying bacteria was composed of 1.0 g/L KNO_3_, 8.5 g/L sodium citrate, 1.0 g/L L-asparagine, 1.0 g/L KH_2_PO_4_, 1.0 g/L MgSO_4_·7H_2_O, 0.2 g/L CaCl_2_·6H_2_O, 0.05 g/L FeCl_3_·6H_2_O, and 0.1% (volume ratio) of the 1% (ratio of weight/volume) alcoholic dissolved bromothymol blue (BTB). The final pH of the DM was adjusted to 7.0. The solid GN was made by adding 2% of agar powder to the liquid GN.

Luria-Bertani broth medium (LB): The LB broth liquid medium consisted of 10 g/L tryptone, 10 g/L yeast extract and 5 g/L NaCl, and 2% (g/L) of agar powder was added to LB broth liquid medium when solid plate medium was needed.

HNM medium consisted of the following components (per liter): 2.87 g of C_6_H_5_Na_3_O_7_, 0.24 g of (NH_4_)_2_SO_4_, 50 mL of HNM trace elements solution. The HNM trace elements solution contained (per liter): 5 g of K_2_HPO_4_ or 6.5 g of K_2_HPO_4_·3H_2_O, 2.5 g of MgSO_4_·7H_2_O and NaCl, 0.05 g of FeSO_4_·7H_2_O and MnSO_4_·4H_2_O [25].

DM medium consisted of the following components (per liter): 2.87 g of C_6_H_5_Na_3_O_7_, 0.36 g of KNO_3_ (DM1) or 0.25 g of NaNO_2_ (DM2), 0.2 g of MgSO_4_·7H_2_O, 1.5 g of KH_2_PO_4_, 10.55 g of Na_2_HPO_4_·12H_2_O or 5.3 g of Na_2_HPO_4_·2H_2_O, 2 mL of DM trace elements solution. The DM trace elements solution contained (per liter): 50 g of EDTA-Na_2_, 2.2 g of ZnSO_4_·7H_2_O, 5.5 g of CaCl_2_, 5.06 g of MnCl_2_·4H_2_O, 5.0 g of FeSO_4_, 1.57 g of CuSO_4_·5H_2_O, and 1.6 g of CoCl_2_·6H_2_O.

All media were sterilized for 30 min at 0.11–0.15 MPa and 121 °C.

### 2.2. Isolation of Potential HN-AD Microorganisms

The method for isolating HN-AD microbes has been described previously [26]. Briefly, an aliquot of water sample from Liangshui River (39.90° N 116.22° E) in Beijing, China was inoculated into 250 mL enrichment medium and incubated at 30 °C with shaking (150 rpm). A total of 25 mL cell culture was replaced with an equal volume of fresh enrichment medium every 48 h for 30 d and a following replacement with 25 mL of fresh denitrifying medium every 24 h for 14 d. Then, 1 mL of the cultivated medium was diluted (10^−1^) with sterilized distilled H_2_O and spread on solid screen medium with the pH indicator bromothymol blue at 30 °C. After cell colonies formed, the blue ones primitively confirmed as aerobic denitrifiers were picked up and incubated individually in liquid screen medium at 30 °C with shaking (150 rpm). Luria-Bertani (LB) broth medium was used to preserve the isolates at 4 °C.

### 2.3. Identification of Isolate W30

Isolate W30 was purified through the plate streaking method on LB solid medium at 30 °C. Cells were fixed with 2.5% glutaraldehyde (pH 6.8) at 4 °C overnight and dehydrated for 20 min each in an ascending ethanol dehydration series (30, 50, 70, 85, 90, and 100%). Cells were dried through a CO_2_ critical point dryer (HCP-2, Hitachi, Tokyo, Japan) and coated with gold (E-1010, Hitachi, Tokyo, Japan). Cell morphology was observed using a scanning electron microscope (SEM, JSM-5800, Hitachi, Tokyo, Japan) at an accelerating voltage of 15 kV. The 16S rRNA gene of W30 was amplified using the primer pair 27F/1492R [27] and sequenced by RuiBiotech company (Beijing, China). The obtained sequence (1353 bp) was aligned using an in-house software CExpress by the company. The phylogenetic tree of W30 was constructed by MEGA 7.0 [28] based on the neighbor-joining method with the partial sequenced 16S rRNA gene of W30 and that of known HN-AD strains after the phylogeny of W30 was determined through the Basic Local Alignment Search Tool program (BLAST) at https://blast.ncbi.nlm.nih.gov/Blast.cgi/ (accessed on 1 November 2021). The information for denitrification genes was collected from Kyoto Encyclopedia of Genes and Genomes (KEGG) database at https://www.genome.jp/kegg/ (accessed on 1 November 2021).

### 2.4. Characterization of the Aerobic N Removal Capacity and the Constraints

#### 2.4.1. Amplification and Identification of HN-AD Related Genes

HN-AD related genes including *napA*, *narZ*, *narH*, *nirK*, *nirS*, *norB* and *nosZ* which were supposed in the genome of W30 were amplified with corresponding primer pairs (Table S1). The PCR mixture (30 μL) was composed of 15 μL 2×EasyTaq PCR SuperMix (RuiBiotech, Beijing), 1 μL of each primer (10 μM), 2 μL DNA template, and 11 μL ddH_2_O. The PCR was carried out as follows: pre-denaturation at 94 °C for 5 min, 35 cycles of denaturation at 94 °C for 30 sec, annealing for 30 sec at 61 °C for *narZ*, at 59 °C for *narH*, at 60 °C for *napA*, at 55 °C for *nirK*, at 53 °C for *nirS*, at 57 °C for *norB*, at 56 °C for *nosZ*. The elongation was at 72 °C for 1 min with a final extension at 72 °C for 7 min. The PCR products were analyzed and purified by 1% agarose gel electrophoresis and sequenced by RuiBiotech company (Beijing, China).

#### 2.4.2. Nitrogen Removal Capacity

A heterotrophic nitrification medium (HNM) and two aerobic denitrification media (DM1 and DM2) were prepared as basic media for characterizing W30′s N removal capacity under oxic conditions [8,29,30]. Ammonium sulfate was the sole N species in HNM, whereas potassium nitrate and sodium nitrite were the N sources in DM1 and DM2, respectively. The initial inorganic N concentration was set at 50 mg/L with a C/N ratio of 16:1 by using 800 mg/L carbon from sodium citrate. The detailed components of HNM, DM1, and DM2 are described in the supplementary information. The initial pH of all media was set at 7.0. An aliquot (5 mL) of W30 cell suspension at the exponential phase was inoculated into 500 mL HNM, 500 mL DM1, and 500 mL DM2 in a 1 L Erlenmeyer flask, respectively. The initial OD_600_ value of each mixture was determined by a spectrometer (VIS-7220N, Beijing Beifen-Ruili Analytical Instrument, Beijing, China). The initial OD_600_ values were 0.148 ± 0.005 for HNM, 0.151 ± 0.007 for DM1, and 0.153 ± 0.004 for DM2. The flasks were incubated at 30 °C for 24 h with shaking at 150 rpm to provide 6.1 mg/L dissolved oxygen in the system. OD_600_, NH_4_^+^-N, NO_3_^−^-N, NO_2_^−^-N, dissolved oxygen (DO), temperature, and Chemical Oxygen Demand (COD) were assayed every 3 h. Total nitrogen (TN) and dissolved total nitrogen (DTN) were tested at 0 h and 24 h, respectively. Except for OD_600_ and TN, the rest of the parameters were measured by filtering each mixture with a 0.45 μm filter (ANPEL, Shanghai, China). NH_4_^+^-N was measured by the colorimetric method with Nessler’s reagent at 420 nm. NO_3_^−^-N was determined by the phenoldisulfonic acid method. NO_2_^−^-N was analyzed by the colorimetric method with N-(1-naphthalene)-diaminoethane. TN and DTN were estimated using the alkaline potassium persulfate digestion-ultraviolet spectrophotometric method (DRB200, Hach, Loveland, CO, USA). COD was determined using the dichromate method. Methods for chemical quantification were referred to standard methods [31] if not otherwise mentioned.

#### 2.4.3. C/N Ratio

A series of C/N ratios (1–20) based on DM1 were investigated to illustrate their effects on W30′s NO_3_^−^-N removal capacity under oxic condition. The required C/N ratios were achieved by changing the content of sodium citrate in the system. The initial NO_3_^−^-N concentration was set as 50 mg/L. The initial pH was 7.0. The initial inoculation volume was 1% (*v*/*v*). The cell culture was incubated at 30 °C for 24 h with shaking (150 rpm). Samples were taken and tested at 0 h and 24 h.

#### 2.4.4. Carbon Sources

Five carbon sources including sucrose, sodium citrate, glucose, sodium acetate, and sodium bicarbonate based on DM1 were investigated to illustrate the effects of different carbon sources on W30′s NO_3_^−^-N removal capacity under oxic condition. The initial NO_3_^−^-N concentration was set as 50 mg/L. The C/N ratio was adjusted to 8. The initial pH was 7.0. The initial inoculation volume was 1% (*v*/*v*). The cell culture was incubated at 30 °C for 24 h with shaking (150 rpm). Samples were taken and tested at 0 h and 24 h.

#### 2.4.5. Dissolved Oxygen

Six levels of dissolved oxygen (1.8, 3.1, 4.2, 5.9, 6.1, and 6.2 mg/L) based on DM1 were investigated to illustrate their effects on W30′s NO_3_^−^-N removal capacity under different DO concentrations. The different levels of dissolved oxygen in the media were achieved using various rotation speeds (from 30 rpm to 180 rpm) of a thermostat vibrating incubator (MQL-61R, Shanghai Minquan Instrument Co., Ltd., Shanghai, China) [11,32]. The initial NO_3_^−^-N concentration was set as 50 mg/L. The C/N ratio was adjusted to 8. The initial pH was 7.0. The initial inoculation volume was 1% (*v*/*v*). The cell culture was incubated at 30 °C for 24 h. Samples were taken and tested at 0 h and 24 h.

#### 2.4.6. Initial pH

A series of initial pH values (3–12) based on DM1 were investigated to illustrate their effects on W30′s NO_3_^−^-N removal capacity under oxic condition. The required pH values were achieved by adjusting the buffering ratio of 0.5 mol/L NaOH and 0.5 mol/L HCl in the system. The initial NO_3_^−^-N concentration was set as 50 mg/L. The C/N ratio was adjusted to 8. The initial inoculation volume was 1% (*v*/*v*). The cell culture was incubated at 30 °C for 24 h with shaking (150 rpm). Samples were taken and tested at 0 h and 24 h.

#### 2.4.7. Temperature

A series of temperatures (15–45 °C) based on DM1 were investigated to illustrate their effects on W30′s NO_3_^−^-N removal capacity under oxic condition. The temperature range (15–45 °C) with a 5 °C ascending was achieved by setting the incubation temperature of the thermostat vibrating incubator (MQL-61R, Shanghai Minquan Instrument Co., Ltd., Shanghai, China). The initial NO_3_^−^-N concentration was set as 50 mg/L. The C/N ratio was adjusted to 8. The initial pH was 7.0. The initial inoculation volume was 1% (*v*/*v*). The cell culture was incubated for 24 h with shaking (150 rpm). Samples were taken and tested at 0 h and 24 h.

#### 2.4.8. Inoculation Volume

A series of inoculation volumes based on DM1 were investigated to illustrate their effects on W30′s NO_3_^−^-N removal capacity under oxic conditions. The different inoculation volumes were achieved by adjusting the amount of the cell culture amendment. The series of inoculation volume was set as 0.1%, 0.2%, 0.5%, 1.0%, and 1.5% (*v*/*v*). The initial NO_3_^−^-N concentration was set as 50 mg/L. The C/N ratio was adjusted to 8. The initial pH was 7.0. The cell culture was incubated at 30 °C for 24 h with shaking (150 rpm). Samples were taken and tested at 0 h and 24 h.

### 2.5. Membrane Bioreactor (MBR) Effluent Assay

An assembled MBR with a biological treatment unit and a membrane unit for separation (pore size: 10 nm) was applied for the effluent assay. The aim of this study was to evaluate the feasibility of using isolate W30 for removing high N content wastewater after MBR treatment when the primary N removal efficiency was low. The influent was domestic wastewater. The daily wastewater treatment capacity of this MBR was about 1.62 m^3^. Continuous aeration (two hours) was followed by an hour of rest. The NO_3_^−^-N concentration in the effluent of the MBR was 58.65 ± 1.34 mg/L. Four treatments were conducted with the effluent to explore W30′s N removal capacity under oxic condition (6.1 mg/L DO) including (i) blank control (no W30 and carbon amendment); (ii) 1% (*v*/*v*) W30 suspension inoculation; (iii) 1% (*v*/*v*) W30 suspension inoculation and sodium citrate amendment, up to 400 mg/L carbon in the system; (iv) 1% (*v*/*v*) W30 suspension inoculation and sodium citrate amendment, up to 800 mg/L carbon in the system. Each treatment had three replicates. The incubations were carried out at 30 °C for 24 h with shaking (150 rpm). Samples were taken and tested at 0 h and 24 h.

### 2.6. Data Analysis and Statistic

The removal efficiency (RE) and the removal rate (RR) of N compounds under different conditions were calculated according to the following formula [11]:

$$\mathrm{Removal}\;\mathrm{efficiency}\;\left(\%\right)=\left(C_{0\;h}-C_{t\;h}\right)/C_{0\;h}\times 100\%$$

$$\mathrm{Removal}\;\mathrm{rate}\;\left(\mathrm{mg}\cdot L^{-1}\cdot h^{-1}\right)=\left(C_{0\;h}-C_{t\;h}\right)/t$$

In which, C_0 h_ and C_t h_ were the initial and final concentrations of each N compound such as NH_4_^+^-N, NO_3_^−^-N, and NO_2_^−^-N at 0 h and t h, respectively.

The cellular N content (biomass produced) was calculated by subtracting cellular-N at 0 h from cellular-N at 24 h [11,33]:

$${\mathrm{Cellular}\;\mathrm{nitrogen}}_{0\;h}\;\left(\mathrm{mg}/L\right)={\mathrm{TN}}_{0\;h}-{\mathrm{DTN}}_{0\;h}$$

$${\mathrm{Cellular}\;\mathrm{nitrogen}}_{24\;h}\;\left(\mathrm{mg}/L\right)={\mathrm{TN}}_{24\;h}-{\mathrm{DTN}}_{24\;h}$$

$$\mathrm{Cellular}\;\mathrm{nitrogen}\;\left(\mathrm{mg}/L\right)={\mathrm{Cellular}\;\mathrm{nitrogen}}_{24\;h}-{\mathrm{Cellular}\;\mathrm{nitrogen}}_{0\;h}$$

Gaseous N content (N loss) was calculated by subtracting TN at 24 h from TN at 0 h as the following formula, or, the produced Gas-N can be also calculated from the parameter DTN:

$$\mathrm{Gaseous}\;\mathrm{nitrogen}\;\left(\mathrm{mg}/L\right)={\mathrm{TN}}_{0\;h}-{\mathrm{TN}}_{24\;h}$$

$$\mathrm{Gaseous}\;\mathrm{nitrogen}\;\left(\mathrm{mg}/L\right)={\mathrm{DTN}}_{0\;h}-\left({\mathrm{DTN}}_{24\;h}+\mathrm{Cellular}\;\mathrm{nitrogen}\right)$$

The denitrification and assimilation efficiency of W30 on N transformation was calculated by the following formula:

$$\mathrm{Assimilation}\;\mathrm{efficiency}\left(\%\right)=\mathrm{Cellular}\;\mathrm{nitrogen}/{\mathrm{DTN}}_{0\;h}\times 100\%$$

$$\mathrm{Denitrification}\;\mathrm{efficiency}\;\left(\%\right)=\mathrm{Gaseous}\;\mathrm{nitrogen}/{\mathrm{DTN}}_{0\;h}\times 100\%$$

TN removal efficiency was estimated by summing denitrification and assimilation efficiency as the following formula:

Total nitrogen removal efficiency (%)=Assimilation efficiency+Denitrification efficiency 

One-way analysis of variance (ANOVA) with Tukey method was used to evaluate the statistical significance of environmental constraints on W30′s N removal capacity under oxic condition. Data are shown as mean ± standard deviation.

### 2.7. Data Availability

The sequenced partial 16S rRNA gene of *Pannonibacter* sp. isolate W30 was deposited in GenBank under accession number KT380575.1. The sequenced functional gene series *nosZ*, *nirK*, *norB*, *narZ*, and *narH* were deposited in GenBank under accession numbers from OK431598 to OK431602.

## 3. Results

### 3.1. Isolation and Identification of Aerobic Heterotrophic Nitrogen Removal Bacterial Isolate W30

**Figure 1:**
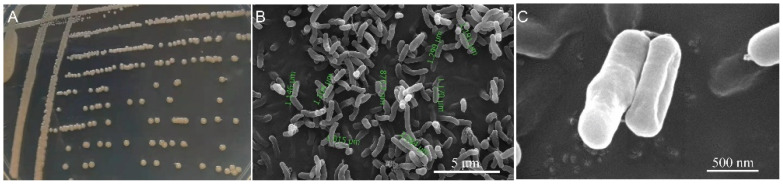
Morphology of isolate W30: (**A**) colonies on LB solid medium; (**B**) scanning electron micrograph of W30 cells. Green marks showed the measured cell lengths; (**C**) detailed morphology of individual W30 cells.

A total of 13 potential HN-AD bacteria were isolated from the 10^−1^ dilution of the cultured Liangshui River sample. One of the aerobic denitrifiers, isolate W30, could form a yellowish, circular, and convex colony with a smooth surface and entire edge on a LB plate. The colony was opaque and sticky (Figure 1A). The cell of W30 showed an average length of 1.24 ± 0.37 μm. Cells were non-spore-forming rods (Figure 1B,C).

**Figure 2:**
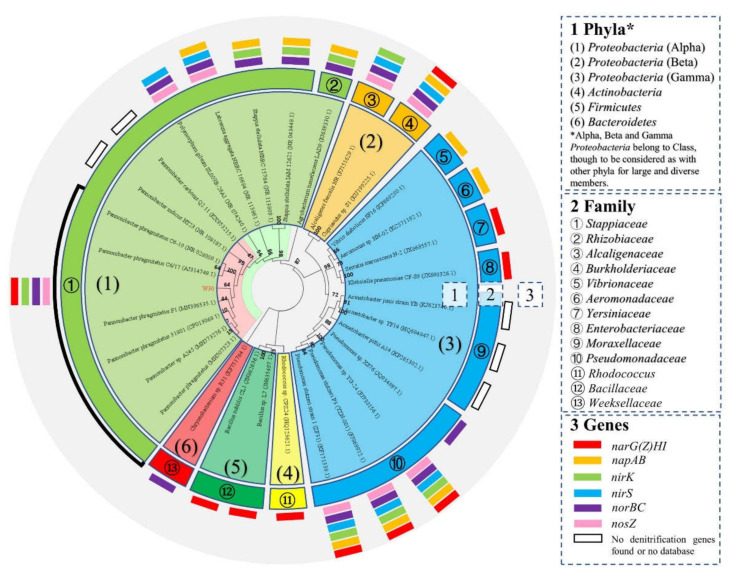
Phylogenetic relationship of isolate W30 within the Bacterial domain based on comparison of partial 16S ribosomal RNA gene sequences. W30′s sequence was aligned to representative sequences from the GenBank databases. Phylogenetic analysis was performed with the MEGA software. The phylogenetic tree was constructed based on 16S ribosomal RNA genes in MEGA by evolutionary distance (neighbor-joining). The information on known functional genes associated with the representative sequences were added in the tree. Accession numbers shown for the comparison sequences were obtained from GenBank. Bootstrap value was 1000 replications. The red rectangle denotes the alpha subunit for nitrate reductase I (*narG*) and nitrate reductase II (*narZ*) for Gram-positive and -negative bacteria with other subunits.

The sequenced partial 16S rRNA gene of W30 (1353 bp) was nearly identical (99.78%) to that of the type strain *Pannonibacter phragmitetus* C6-19 (NR_028009.1) (Figure 2), which belongs to the class *Alphaproteobacteria*. The phylogenetic tree based on evolutionary distance (neighbor-joining) showed that the HN-AD microorganisms were quite diverse, distributed among at least 13 different families. The genus *Pannonibacter* comprised several species such as *P*. *phragmitetus*, *P*. *indicus*, and *P*. *carbonis*. Although isolate W30 was grouped within *Pannonibacter* and close to *Pannonibacter phragmitetus*, it detached slightly from the internal clusters of the genus. As observed in the phylogenetic tree (Figure 2), outside the genus *Pannonibacter*, the closest species to W30 was *Polymorphum gilvum*, which is a potential HN-AD bacterium belonging to the *Alphaproteobacteria* class that is able to reduce nitrite to N_2_ based on the related functional genes in the KEGG database. Therefore, the isolate W30 was primitively designated as *Pannonibacter* sp. W30. Furthermore, the genetic relationship between W30 and other reported typical HN-AD bacteria were also exhibited, such as *Agrobacterium tumefaciens* LAD9 [34], *Alcaligenes faecalis* NR [35], *Klebsiella pneumoniae* CF-S9 [36], *Acinetobacter junii* strain YB [25,37], *Pseudomonas stutzeri* YZN-001 [20], and *Bacillus methylotrophicus* strain L7 [38]. Various denitrification-related genes were detected in the genomes of known HN-AD microbes in the phylogenetic tree including *narG(Z)HI*, *napAB*, *nirK*, *nirS*, *norBC*, and *nosZ*. An entire series of reduction genes targeting substances from nitrate to N_2_O were detected in *Stappiaceae* (https://www.genome.jp/pathway/pphr00910/, accessed on 1 November 2021), *Burkholderiaceae* (https://www.genome.jp/pathway/reh00910/, accessed on 1 November 2021), and *Pseudomonadaceae* (https://www.genome.jp/pathway/psz00910/, accessed on 1 November 2021), which belong to *Proteobacteria* only. Other HN-AD microbes showed no full capacity to reduce all the intermediates in denitrification.

### 3.2. Isolate W30′s Capacity on N Removal and Constraints under Oxic Condition

#### 3.2.1. The Aerobic Denitrification Potential of Isolate W30

**Figure 3:**
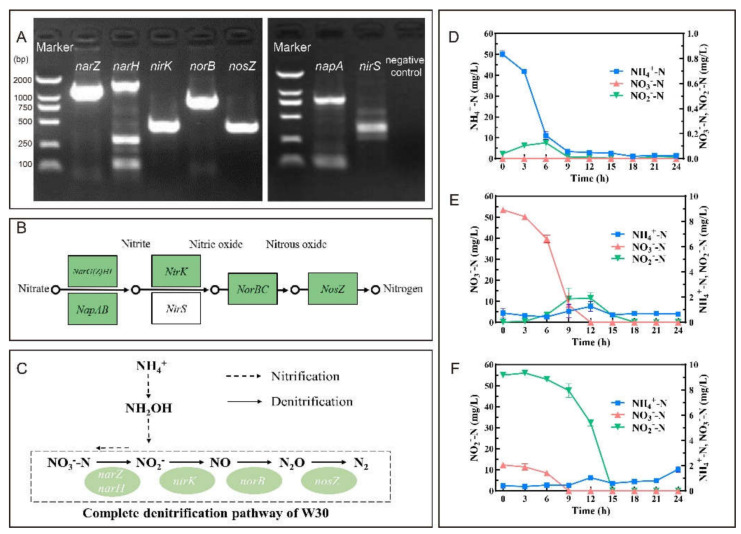
Isolate W30′s potential and capacity on N removal. (**A**): The amplification of denitrification genes of isolate W30. (**B**): Reference denitrification pathway of *Pannonibacter phragmitetus* 31801 (GenBank: CP013068) from KEGG database (www.genome.jp/pathway/pphr00910/, accessed on 1 November 2021). (**C**): Proposed denitrification pathway of isolate W30. Nitrogen removal characteristic of isolate W30 in media of (**D**) HNM, (**E**) DM1, and (**F**) DM2. Symbols: ■, NH_4_^+^-N; ▲, NO_3_^−^-N; ▼, NO_2_^−^-N. Values represent the mean ± SD (standard deviation) of three replicates.

The entire series of denitrification genes were successfully amplified from W30′s genome (Figure 3A). However, the amplification results of the periplasmic nitrate reductases gene *napA* and nitrite reductase *nirS* were negatively supported by further sequencing. The results were in line with the N metabolism pathway of *Pannonibacter phragmitetus* 31801 (GenBank: CP013068) shown in KEGG (Figure 3B). Therefore, a denitrification pathway (NO_3_^−^-N → NO_2_^−^-N → NO → N_2_O → N_2_) of *Pannonibacter* sp. W30 under oxic conditions was proposed (Figure 3C).

#### 3.2.2. HN-AD Performance of Isolate W30

Isolate W30 could heterotrophically remove NH_4_^+^-N in HNM with an average rate of 5.18 mg/L/h in 9 h (Figure 3D). The removal rate was 1.34-fold higher than that of *Ochrobactrum anthropic* LJ81 (3.85 mg/L/h) [32] and 2.26-fold higher than that of *Vibrio diabolicus* SF16 (2.29 mg/L/h) [15]. The removal efficiency of NH_4_^+^-N was 97.11% with a linear average rate of 10.21 mg/L/h during 3-6 h. At the same time, a NO_2_^−^-N anomaly around 0.105 ± 0.005 mg/L (3 h) and 0.127 ± 0.058 mg/L (6 h) appeared but rapidly decreased therefrom. In addition, no obvious amount of NO_3_^−^-N appeared during isolate W30-initiated heterotrophic nitrification.

Isolate W30′s ability of aerobic denitrification was evaluated using DM1 and DM2 with KNO_3_ and NaNO_2_ as the sole N source, respectively. The concentration of NO_3_^−^-N was drastically depleted under the oxic condition from 53.43 ± 0.73 mg/L to a concentration below the detection limit in 12 h (Figure 3E). The linear average removal rate was 10.46 mg/L/h during 6–9 h which was much higher than that of *Klebsiella pneumonia* CF-S9 (8.64 mg/L/h) [30]. Moreover, the removal efficiency could reach 100%. NO_2_^−^-N appeared at 6 h and reached 1.913 ± 0.448 mg/L at 12 h, which was in parallel to the linear depletion of NO_3_^−^-N. Aerobic utilization of NO_2_^−^-N by isolate W30 was also observed (Figure 3F). The linear average depletion rate was 10.77 mg/L/h during 9-12 h and only a trace amount of NO_2_^−^-N remained in the system at 24 h (≤0.016 mg/L). The removal efficiency of NO_2_^−^-N by isolate W30 in 24 h was 99.98%.

Through HN-AD, isolate W30 achieved high TN removal efficiency with various N sources (Table 1). When (NH_4_)_2_SO_4_ was the sole N source in HNM, the highest TN removal efficiency could reach 91.34%. Relative to NO_3_^−^-N (8.74%) and NO_2_^−^-N (10.31%), more gaseous N was prone to occur when NH_4_^+^-N (26.64%) was the N source. Assimilation was the dominant driver for N depletion in all the systems. There is no marked difference in the denitrification of KNO_3_ and NaNO_2_ in DM1 and DM2.

#### 3.2.3. NO_3_^−^-N Removal Constraints of Isolate W30

**Figure 4:**
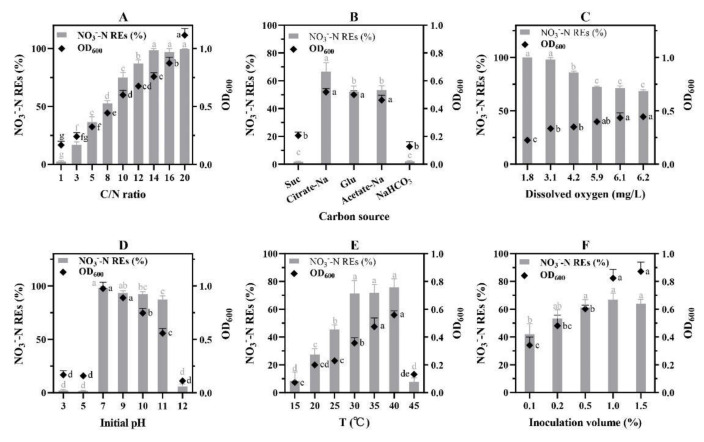
Species-specific constraints on NO_3_^−^-N removal mediated by W30. (**A**) C/N ratio; (**B**) carbon sources, Suc and Glu denoted sucrose and glucose, respectively; (**C**) dissolved oxygen; (**D**) initial pH; (**E**) temperature; (**F**) inoculation volume. Values with different letters indicate being significantly different at *p* < 0.05.

The investigation on the effect of C/N ratio showed that the higher the ratio, the greater the N removal efficiency, until the ratio reached 14 (Figure 4A). The isolate W30 was able to function normally with C/N ratios ranging from 3 to 20. With a C/N of 3, isolate W30 was able to remove 16.96 ± 2.52% NO_3_^−^-N. When the ratio was 1, little N (2.36 ± 0.77%) was removed. As the C/N ratio increased to 14, W30 achieved the greatest NO_3_^−^-N removal (98.62 ± 1.40%). Increasing the C/N ratio from 14 did not enhance NO_3_^−^-N removal efficiency while cell density continually increased.

Different carbon sources resulted in different NO_3_^−^-N removal efficiencies for isolate W30 (Figure 4B). The highest removal efficiency was achieved by using sodium citrate, which was 66.57 ± 6.53%. Glucose and sodium acetate showed no statistical significance on NO_3_^−^-N removal, which were 52.20 ± 4.0% and 53.27 ± 3.19%, respectively. Sucrose and sodium bicarbonate were not the preferential carbon sources for W30 to reduce NO_3_^−^-N in 24 h. The NO_3_^−^-N removal efficiencies were only 1.80 ± 0.46% and 2.10 ± 0.46%, respectively.

Six dissolved oxygen concentrations were achieved by adjusting the rotation speed from 30 to 180 rpm representing 1.8, 3.1, 4.2, 5.9, 6.1, and 6.2 mg/L DO (Figure 4C). The highest NO_3_^−^-N removal efficiency was achieved at anoxic/hypoxic conditions (1.8 and 3.1 mg/L DO) with no statistical significance. Following that, the NO_3_^−^-N removal efficiency decreased as dissolved oxygen concentrations increased from hypoxic condition to oxic condition (5.9 mg/L DO) significantly. Finally, a medium NO_3_^−^-N removal efficiency occurred at oxic conditions (DO ≥ 5.9 mg/L).

Isolate W30 could not reduce NO_3_^−^-N at low pH (Figure 4D). The NO_3_^−^-N removal efficiencies were only 2.21 ± 0.87% and 1.67 ± 0.46% at pH 3 and 5, respectively. The optimal pH range allowing isolate W30 to proliferate was from 7 to 11 when the maximum NO_3_^−^-N removal efficiency reached 98.35 ± 1.03% at neutral pH.

A linear relationship between temperatures and NO_3_^−^-N removal efficiencies was observed from 15 °C to 30 °C (Figure 4E). The highest NO_3_^−^-N removal efficiency was achieved at 40 °C. However, the nitrate removal ability decreased markedly when W30 was cultured at 45 °C (7.80 ± 2.90%) and 15 °C (8.45 ± 6.31%).

NO_3_^−^-N removal efficiencies were proportional to the initial inoculation volumes from 0.1% to 0.5%, whereas no statistical significance was observed from 0.5% to 1.5% (Figure 4F).

### 3.3. Pilot MBR Effluent Test

The major N species in the effluent of the pilot MBR was NO_3_^−^-N (58.65 ± 1.34 mg/L) with a trace amount of NH_4_^+^-N and NO_2_^−^-N. Isolate W30 showed a high capacity for NO_3_^−^-N removal in the effluent under oxic condition if a sufficient carbon source was supplied (Table 2). A total of 99.3% NO_3_^−^-N could be reduced in 24 h with 800 mg/L carbon from sodium citrate.

## 4. Discussion

The development of new wastewater treatment technologies has been influenced by the discovery that HN-AD microorganisms can simultaneously nitrify NH_4_^+^-N and denitrify NO_3_^−^-N. A number of HN-AD mechanisms and pathways have been identified, including denitrification mechanisms such as nitrate–nitrite reduction or hydroxylamine reduction, and others as incomplete N removal and organic N removal [7]. There is an increasing recognition that the HN-AD process is species-specific. The characteristics and constraints of diverse HN-AD microorganisms play a pivotal role in N removal efficiency. Among all the known HN-AD microbes, several potential *Alphaproteobacteria* HN-AD microbes have been identified, such as *Polymorphum gilvum* SL003B-26A1, *Labrenzia aggregata* NBRC 16684, *Stappia stellulata* NBRC15764, *Stappia stellulata* LAM 12621, and *Agrobacterium tumefaciens* LAD9. With the exception of *norBC*, which was found in most *Alphaproteobacteria* HN-AD strains, all other reductase genes involved in denitrification differed among *Alphaproteobacteria* species. In our study, the negative amplification of *napA* of the periplasmic nitrate reductases, a specific enzyme in aerobic denitrification, indirectly verified that membrane-bound nitrate reductases might prevail in *Pannonibacter*. The membrane-bound nitrate reductase gene *narG* was amplified from both W30 and *Pannonibacter phragmitetus* B1 [39]. Since O_2_ inhibits the activity of membrane-bound nitrate reductases [40], W30′s ability of NO_3_^−^-N removal under oxic conditions may involve additional mechanisms. The nitrite reductase gene *nirK* (467 bp) and the nitrous oxide reductase gene *nosZ* (447 bp) were successfully amplified, indicating that W30 could use nitrite as an intermediate electron acceptor for aerobic denitrification [39] and nitrous oxide could be reduced to N_2_ during the process. It is noteworthy that among a few known HN-AD microbes belonging to *Alphaproteobacteria* including *Paracoccus*, *Pannonibacter*, and *Agrobacterium*, *Pannonibacter* shows an advantage to develop a felicitous technology for the treatment of N-laden wastewater at the full-scale level. According to the N metabolism pathway in KEGG, the type strain *Agrobacterium* sp. RAC06 has no *nosZ* in its genome, which may lead to the accumulation of nitrous oxide, a major scavenger of stratospheric ozone. In addition, *nirS* rather than *nirK* is in the genome of *Paracoccus denitrificans* [8]. *nirS* encodes a homodimer cytochrome cd1-containing nitrite reductase while *nirK* encodes a copper-containing nitrite reductase [41,42]. The iron-based nitrite reductase *nirS* may be sensitive to environmental pH, which limits its application in wastewater treatments. Indeed, it was reported that a greater loss in *nirS* abundance occurred when the environmental pH was below 4.7 [43].

Isolate W30 could remove ammonia with a high rate and efficiency (97.11%) by two processes, assimilation into cellular nitrogen and aerobic denitrification (AD). Furthermore, nitrite was found to be a nitrification intermediate during this latter process as it was produced in trace amounts at the initial 6 h and then quickly consumed. This indicated that isolate W30 could transform ammonia to nitrite by heterotrophic nitrification (HN) and then use it for aerobic denitrification (AD). In parallel, no obvious amount of nitrate was detected during isolate W30-mediated HN. However, it was reported that a known HN-AD bacterium *Acinetobacter* sp. ND7 produced 4.7 mg/L of nitrate from ammonia at the initial 8 h of heterotrophic nitrification, but it delayed till 24 h to consume it completely [11]. Therefore, it seems that W30 can rapidly transform ammonia under oxic conditions by HN-AD with the advantage of non-accumulate nitrate in the environment. Isolate W30′s ability of aerobic denitrification (AD) was also evaluated and it was a half to that reported for other HNAD microorganisms when ammonia was the initial N source [11]. However, removal efficiency of nitrite and nitrate by isolate W30 in 24 h (99.98% and 100%, respectively) was mainly obtained through assimilation. Thus, we have found that when KNO_3_ and NaNO_2_ were the initial N sources, isolate W30 was apt to store them as cellular nitrogen.

Despite most *Alphaproteobacteria* being oligotrophs, isolate W30 seemed to benefit from high C/N ratios during aerobic NO_3_^−^-N removal. This result is in line with that of other aerobic denitrifiers such as *Citrobacter diversus* [44] and *Cupriavidus* sp. S1 [30]. An earlier study found that oligotrophic and eutrophic bacteria were interchangeable, depending on both the specific nutrients available and their concentrations [45]. In this context, isolate W30, an *Alphaproteobacteria*, could adapt to a broad and flexible range of C/N ratios. Moreover, W30 was verified a strict heterotroph through the NaHCO_3_ test. Meanwhile, sucrose was verified not a favorite carbon source for W30. W30′s aerobic N removal efficiency decreased as dissolved oxygen concentration increased. Similarly, an aerobic denitrification bacterium *Citrobacter diversus* showed the highest denitrification rate when dissolved oxygen was low [44]. For isolate W30, however, a significant NO_3_^−^-N removal efficiency could still be achieved even at oxic conditions, indicating W30 is an aerobic bacterium that could tolerate high O_2_ concentrations. In addition, W30 exhibited a series of high NO_3_^−^-N removal efficiencies from neutral to alkaline pHs. The result is consistent with that of known HN-AD microbes such as *Pannonibacter phragmitetus* F1 [46] and a strain of *Pannonibacter* isolated from a Hungarian soda lake [47]. It is believed that acidic pH may affect free ammonia and ammonia monooxygenase in the environment, although explicit mechanisms need to be further explored [38]. Moreover, high NO_3_^−^-N removal efficiencies up to 75.73 ± 6.23% were achieved by W30 at temperatures from 30 °C to 40 °C. As a comparison, the NO_3_^−^-N removal efficiency of *Acinetobacter* sp. ND7 reached only 6.69 ± 0.66% at 40 °C [11]. Our result also verified that 0.5% inoculation volume (initial OD_600_ = 0.305) was sufficient for initiating the NO_3_^−^-N removal process. Furthermore, *Pannonibacter* sp. W30 achieved nearly full NO_3_^−^-N removal in effluent from the pilot MBR. The C/N ratio, however, seemed critical for isolate W30 to tackle high N content wastewater at the full-scale level.

## 5. Conclusions

An aerobic heterotrophic nitrogen removal *Alphaproteobacteria Pannonibacter* sp. W30 was isolated, identified, and characterized in this study. A potential HN-AD pathway of W30 was proposed. The effect of environmental constraints on aerobic NO_3_^−^-N removal was investigated. Based on the results, *Pannonibacter* W30 was proved to be able to perform N removal from water environments with high inorganic N removal efficiency. The study verified that *Pannonibacter* sp. W30 contains *nirK*, *norB,* and *nosZ* genes which encode a nitrite reductase, a nitric oxide reductase, and a nitrous oxide reductase. These enzymes can transform NO_2_^−^-N to NO, N_2_O, and N_2_, avoiding NO_2_^−^-N accumulation in the process. *Pannonibacter* sp. W30 also has a copper-containing nitrite reductase *nirK*, a relatively robust enzyme under low pH. Our results suggest that although *Pannonibacter* sp. W30 could be able to conduct aerobic denitrification, dissolved oxygen is still a limiting factor affecting this process. As an aerobic heterotrophic nitrogen removal bacterium, *Pannonibacter* sp. W30 adapts to a broad range of C/N ratios in wastewaters, which may be feasible for developing novel HN-AD technologies.

## Figures and Tables

**Table 1:** W30′s N removal capacity under oxic condition.

N-Source (mg/L)	Incubation Time (h)	DTN (mg/L)	Gaseous-N (mg/L)	Cellular-N (mg/L)	Denitrification Efficiency (%)	Assimilation Efficiency (%)	TN RE (%)
NH_4_^+^-N (50.03 ± 1.69)	0 h	50.03 ± 1.69	-	-	-	-	-
	24 h	4.33 ± 2.52 ^a^	13.33 ± 0.58	32.37 ± 3.22	26.64	64.70	91.34
NO_3_^−^-N (53.43 ± 0.73)	0 h	53.43 ± 0.73	-	-	-	-	-
	24 h	6.33 ± 1.15 ^b^	4.67 ± 2.89	42.43 ± 2.46	8.74	79.41	88.15
NO_2_^−^-N (55.00 ± 0.50)	0 h	55.00 ± 0.50	-	-	-	-	-
	24 h	8.67 ± 0.58 ^c^	5.67 ± 1.53	40.67 ± 2.47	10.31	73.95	84.26

^a^. It includes NH_4_^+^-N (1.45 ± 0.03 mg/L); ^b^. NO_3_^−^-N is not detected; ^c^. It includes NO_2_^−^-N (≤0.016 mg/L).

**Table 2:** Batch treatment results of MBR effluent by isolate W30.

Treatment	I	II	III	IV
Outlet water (Effluent)	+	+	+	+
Suspension solution	−	+	+	+
Sodium citrate (400 mg/L C)	−	−	+	−
Sodium citrate (800 mg/L C)	−	−	−	+
Initial NO_3_^−^-N (mg/L)	58.65 ± 1.34			
Final NO_3_^−^-N (mg/L)	53.56 ± 4.25	53.93 ± 2.62	20.35 ± 1.62	0.43 ± 0.06
NO_3_^−^-N removal efficiency	8.7% ^a^	8.0% ^a^	65.3% ^b^	99.3% ^c^

+ means amendment. − means no amendment. Different letters with the values of NO_3_^−^-N removal efficiency indicate a significant difference for *p* < 0.05.

## Data Availability

The data presented in this study are openly available in the NCBI. The accession number has been listed in the article.

## Supplementary Materials

The following are available online at https://www.mdpi.com/article/10.3390/microorganisms10020235/s1, Table S1: Primer pairs related to aerobic denitrification gene amplification.
