# Supplementary material for: Nitrogen Removal Characteristics and Constraints of an Alphaproteobacteria with Potential for High Nitrogen Content Heterotrophic Nitrification-Aerobic Denitrification
Source: Microorganisms. 2022 Jan 21;10(2):235. doi: 10.3390/microorganisms10020235 (PMC8879992; doi:10.3390/microorganisms10020235)
Supplement: Supplementary file 1 [file microorganisms-10-00235-s001.zip › microorganisms-1485224-supplementary.pdf]

Table S1. Primer pairs related to aerobic denitrification gene amplification.

Gene	Primer (5'-3')	References
narG(Z)	GAAGACCGCGGCTGGGAAGA	This study
	CCTTGCCGCCGAAATAGGGG	
narG(H)	GCGCTCAAATCGGCATGGTG	This study
	GCTTGGACGCGCCGAAGA	
napA	TCTGGACCATGGGCTTCAACCA	[1-5]
	ACGACGACCGGCCAGCGCAG	
nirS	CCTAYTGGCCGCCRCART	[6,7]
	CGTTGAACTTRCCGGT	
nirK	ATCATGGT (C/G) CTGCCGCG	[6,8,9]
	GCCTCGATC (A/G) AGTTGTGGTT-3'	
norB	CG (N) GA (R) TT (Y) CT (S) GA (R) CA (R) CC	[10]
	C (R) TA (D) GC (V) CCR (W) AGAA (V) GC	
nosZ	CGYTGTTCMTCGACAGCCAG	[11,12]
	CGSACCTTSTTGCCSTYGCG	
R=A/G, Y=C/T, S=C/G, W=A/T, V=A/C/G, D=A/G/T, N=A/C/G/T		

Reference

1. Tinglin, H., et al., Nitrogen-removal efficiency of a novel aerobic denitrifying bacterium, Pseudomonas stutzeri strain ZF31, isolated from a drinking-water reservoir. Bioresource technology, 2015. 196.
2. Huang, X., et al., Ammonium removal by a novel oligotrophic Acinetobacter sp. Y16 capable of heterotrophic nitrification-aerobic denitrification at low temperature. Bioresour Technol, 2013. 146: p. 44-50.
3. Kong, Q.X., et al., Development and application of a novel and effective screening method for aerobic denitrifying bacteria. FEMS Microbiol Lett, 2006. 260(2): p. 150-5.
4. Bell, L.C., D.J. Richardson, and S.J. Ferguson, Periplasmic and membrane-bound respiratory nitrate reductases in Thiosphaera pantotropha. The periplasmic enzyme catalyzes the first step in aerobic denitrification. FEBS Lett, 1990. 265(1-2): p. 85-7.
5. Bell, L.C., et al., Insertion of transposon Tn5 into a structural gene of the membrane-bound nitrate reductase of Thiosphaera pantotropha results in anaerobic overexpression of periplasmic nitrate reductase activity. J Gen Microbiol, 1993. 139(12): p. 3205-14.
6. Braker, G., A. Fesefeldt, and K.P. Witzel, Development of PCR primer systems for amplification of nitrite reductase genes (nirK and nirS) to detect denitrifying bacteria in environmental samples. Appl Environ Microbiol, 1998. 64(10): p. 3769-75.
7. Velusamy, K. and K.K. Krishnani, Heterotrophic nitrifying and oxygen tolerant denitrifying bacteria from greenwater system of coastal aquaculture. Appl Biochem Biotechnol, 2013. 169(6): p. 1978-92.
8. Wang, Q., et al., Effects of the nitrification inhibitor dicyandiamide (DCD) on N 2 Oemissions and the abundance of nitrifiers and denitrifiers in two contrasting agricultural soils. Journal of Soils and Sediments, 2017. 17(6).
9. Fang, F., S. Chen, and G. Xu, Investigation of Denitrifying Bacteria Communities in Fresh and Aged Municipal Solid Waste Using Nitrite Reductase Genes. Environmental Engineering Science, 2010. 27(11): p. 931-938.
10. Wan, C., et al., Aerobic denitrification by novel isolated strain using NO-(2)-N as nitrogen source. Bioresour Technol, 2011. 102(15): p. 7244-8.
11. Li, X., et al., Characteristics of biofilms and iron corrosion scales with ground and surface waters in drinking water distribution systems. Corrosion Science, 2015. 90.
12. Veraart, A.J., et al., Denitrification in restored and unrestored Danish streams. Ecological Engineering, 2014. 66.
